# Elements of Chemistry, Including the Application of the Science in the Arts

**Published:** 1841-01

**Authors:** 


### Art. VI.

1. *Elements of Chemistry, including the application of the Science in the Arts.* By Thomas Graham, F.R.S. L. & E., Professor of Chemistry in the London University, &c. &c. Parts I.—V.; comprehending *Inorganic Chemistry.—London*, 1837-40. 8vo.
2. *Chemistry of Organic Bodies: Vegetables.* By Thomas Thomson, M.D., F.R.S. L. & E., Professor of Chemistry in the University of Glasgow, &c. &c.—*London*, 1838. 8vo, pp. 1076.
3. *An Introduction to the Study of Chemical Philosophy; being a preparatory View of the Forces which concur to the production of Chemical Phenomena.* By J. Fred. Daniell, F.R.S., Professor of Chemistry in King's College, London, &c. &c.—*London*, 1839. 8vo, pp. 565.
4. *Supplement to the Introduction to the Atomic Theory; comprehending a Sketch of certain Opinions and Discoveries bearing upon the general principles of Chemical Philosophy which have been brought into notice since the publication of that Work. Prefaced by some Remarks on the projected Reforms in Academical Education.* By Charles Daubeny, M.D., F.R.S., &c. &c.; Professor of Chemistry and of Botany in the University of Oxford.—*London*, 1840. 8vo, pp. 62.

We have for some time been intending to offer our readers a brief general account of the recent progress and present state of chemical philosophy, chiefly for the benefit of those who, like ourselves, feel it impossible to devote more time to this engrossing subject than is necessary to keep pace with its generalizations; extending, as these rapidly are, in comprehensiveness, and increasing in certainty. Dr. Daubeny's pamphlet so nearly corresponds in character with the review we had intended to give of the first three works on our list, that we might almost have satisfied ourselves with directing the attention of our readers to its contents. It is evident, however, that the learned author destined it chiefly for a local circulation; and we are sure that we shall not displease him, whilst we confer a benefit on those who would like to receive his concise view in a state of still greater compression, in transferring to our pages copious extracts from his very luminous summary.

In the present article it will be our main object to set before our readers, in the narrowest possible compass, the chief advances in the principles of chemistry, to which the labours of recent years have given occasion. We shall suppose them to possess a general acquaintance with the laws of definite proportions and the atomic theory, as propounded by Dalton; since these are embodied in every systematic work of modern date on this science, and have been made the foundation of all public instruction upon it for some years past. When Dalton had succeeded in obtaining the assent of the scientific world to his generalizations, it was imagined, and not without reason, that the science of chemistry had attained a degree of precision, which might place it on a level with those usually regarded as the exact sciences. It appeared possible to embrace within the compass of a few very simple propositions all the laws which regulate chemical combination; and when, as in the case of organic substances, the phenomena were such as could not be directly deduced from the principles of this theory, it was then thought reasonable to refer such anomalies to the operation of the living principle, that mysterious agent, which, by imparting new properties and new affinities to the inert mass, removed it, as it were, beyond the domain which came under the exclusive jurisdiction of chemical laws. We shall express, in Dr. Daubeny's language, a small number of propositions, which at that time appear to have formed the creed, expressed or understood, of chemists in general, and on which our subsequent remarks will be based.

"1. Matter is composed of a number of particles incapable of further division (or of atoms), bound together by the force of cohesive attraction; and compound bodies are produced by the union of ultimate particles of different kinds of matter possessing an affinity one for the other.

"2. There exists, therefore, a variety of elementary bodies, each of which is distinguished by the possession of certain fixed and unalienable properties, chemical as well as physical; so that the business of the analytical chemist is limited to the determination of the nature of the several elements which enter into the composition of the substance he examines, and of the definite proportion they bear to each other.

"3. The difference between one compound body and another can be referred only to two causes, namely, either to their being formed of different elements, or of the same elements in different proportions; so that all bodies possessing the same chemical composition must be regarded as identical both as to *form* and *nature*—in physical as well as in chemical properties; whilst those which disagree, either in the nature or in the proportion of their component parts, must be expected to differ likewise one from the other in both the above particulars.

"4. Decomposition is caused by the approach of a foreign body, possessing a stronger affinity for one of the ingredients of a compound than that which binds the latter mutually together; so that, in every such case, the decomposing agent may be expected to enter into union with one or other of the elements of the combination which it destroys.

"5. The water, which is found attached to many bodies, is essential to their crystallization, but does not materially affect their intrinsic properties; a salt perfectly divested of water being in no respect to be regarded as chemically different from one which contains it.

"6. Organic bodies have their particles held together partly by what is called the *living principle*—a principle distinct from chemical affinity, and superadded to it; so that the laws which operate upon inorganic matter are, in many instances, suspended by its influence. Hence it is idle to expect that any of the various chemical compounds which result from processes taking place in the living body can be produced by artificial means; they being themselves resolved into their component elements, or into simpler forms of combination, so soon as the sustaining force, the principle of life, is abstracted." (pp. 2, 3.)

Most of the above propositions may be regarded as taken for granted, if not embodied in the atomic theory, as propounded by Dalton. This theory was erected, as our readers will recollect, on the law of definite proportions, which must be considered as his real discovery, and which was pronounced by Sir John Herschel to be, after the laws of mechanics, the most important which the study of nature had disclosed at that period; standing foremost, as it does, among the discoveries of the present age, for the universality of its applications, and the importance of its practical results. It will be found, we believe, that *the essential part* of Dalton's propositions is applicable to all the new discoveries announced in chemistry since its promulgation; so that each succeeding year has added something to the completeness of the evidence upon which it is based, as well as to the extent of the field which its jurisdiction embraces. This distinction it may claim equally with the Newtonian system; to which, in the scale of physical truths, it may not improperly be compared. It cannot be denied, however, that since the period of the reception of the Daltonian *theory*, erected upon those propositions, many new views, involving some fundamental points of theory, have obtained currency amongst chemists; which, though perhaps by no means irreconcilable with that hypothesis, cannot be said to be comprehended under its principles, and which run counter to many of the propositions which have been just quoted as having received a tacit, if not a distinct recognition as appertaining to it. We shall now proceed to notice the more important among these novelties; adverting, in the first instance, to those which simply extend the application of the law of definite proportions, and afterwards discussing those of which the relation to it is less evident.

The two great classes of combinations known as *acids* and *alkalies*, or alkaline *bases*, were long considered the only ones by which *neutral salts* could be formed; all these alkaline bases (with the exception of ammonia), as well as most of the acids, being formed by the union of oxygen with one of the elementary bodies. Thus, the salt known as carbonate of potass is composed of carbonic acid—a compound of carbon and oxygen, and of potass—a compound of potassium and oxygen. The researches of Berzelius and his followers have made it evident that *sulphur* is to be regarded in the same light, and that the *sulphurets* of the different elementary substances have relations, as *acid* and *base*, exactly parallel to those of the oxides. Thus, the bisulphuret of carbon, or the sesquisulphuret of arsenic, forms a *salt* with sulphuret of potassium; which, by the substitution of oxygen for sulphur in *each* ingredient, would form carbonate or arsenite of potass. Similar compounds are formed by the union of the chlorides, iodides, &c. with each other. These have been known as *double* chlorides or iodides; but it is now certain that they are to be regarded as *salts*—one of the ingredients acting as the acid or electro-negative substance, the other as the electro-positive substance or base.

Moreover, it has been shown that two oxides, iodides, or chlorides of the same metal may combine with each other in the relation of acid and base, in accordance with the relative proportions of oxygen, &c. which they contain. Thus, chromic acid, formed of 1 chromium and 3 oxygen, will unite with and be saturated by the sesquioxide of chromium, formed of 1 chromium and $1\frac{1}{2}$ oxygen. Further, two *salts* may unite together in a similar relation, one acting as the electro-positive and the other as the electro-negative ingredient. Thus, sulphate of potass will act as a base to sulphate of alumina (in which the proportion of oxygen is greater), and will form with it a double salt, familiarly known as alum. It is evident that, by the discovery of these facts, the application of the law of definite proportions has received an immense extension.

But this is not all. The study of the compounds of *cyanogen* has led to the discovery that this, though itself a compound body (formed by the union of 2 carbon with 1 nitrogen), acts like a simple or elementary body in all its relations, forming *cyanides*, which are parallel in character to oxides, chlorides, and iodides, and which may stand to each other in the relation of acid and base. Cyanogen, then, is to be regarded as a *compound radical*; that is, as a simple body in its mode of combination, though compounded in its composition. To use the language of the atomic theory, 2 atoms of carbon and 1 of nitrogen together form a single atom or combining equivalent. Now this discovery has opened the way to a series of the most unexpected results, and may be regarded as having afforded the key by which the dark chamber of organic chemistry has been entered and explored. It will be presently seen that such compound radicals are so universally present in organic combinations, that the appellation lately given to this department of the science—the chemistry of compound radicals—would be extremely appropriate, if such combinations were not also to be met with elsewhere. But, as will be presently seen, it is not improbable that the same system prevails in the inorganic world to a much greater extent than has been generally supposed; and that, in fact, our ideas, simple as they appeared, of the union of acids and bases will have to undergo an entire revolution.

In what has been hitherto said, the old phraseology has been employed, because our object has been to indicate the analogy between the compounds of sulphur and cyanogen and those of oxygen. If the long-established view of their composition is changed in regard to one, it will have to be for all alike. We shall now pass on to some of the cases in which it seems pretty well ascertained that compound radicals, of much more complex composition than cyanogen, play the same simple part. The cyanuret of iron will not only combine with the cyanurets of potassium, &c. but also with that of hydrogen—hydrocyanic acid. Now, of such a compound, two views may be taken. It may either be regarded as a salt, like the former, or it may be considered that the whole of the cyanogen in the compound unites with the iron, forming a new radical, which unites with the hydrogen as a simple substance. On the one view, the composition will be thus represented (we avoid the use of symbols for the benefit of our older readers): 2 (hydrogen + cyanogen) serving as the base to (iron + cyanogen). On the other it would be (2 hydrogen) united with (iron + 3 cyanogen). The latter is the opinion of most chemists at the present time; and the existence of a compound radical, ferro-cyanogen, serving as an elementary body, although actually consisting of three, is generally recognized. There is little doubt that cyanogen and sulphur, cyanogen and carbon, and other similar compounds, act as radicals or elementary substances in their combinations. It becomes difficult, then, to assign the limit to this kind of action; and the complex constitution of a body need no longer be regarded as essential by altering its chemical relations.

It will be observed that cyanogen may be regarded as the connecting link between organic and inorganic substances; for, though so simple in its composition, it cannot be produced by the artificial union of its elements, and can only be obtained from substances which have been elaborated by the living system. We must, therefore, consider cyanogen as an organic compound, although so simple in its relations. The facility with which these may be investigated is a great aid to the organic chemist; for, whilst most organic compounds are disposed to separate into their *ultimate* constituents, when submitted to any analytic process, those of cyanogen are much more easily resolved into their *proximate* constituents, by the reunion of which the compound may be readily reproduced. Now it is becoming more and more apparent, that it is only required by the organic chemist that he should *know how* to analyze the substances of which he wishes to ascertain the composition, so as to prevent them from separating at once into their ultimate constituents—to make the *theory* of organic chemistry as simple as that of inorganic; and, with certain data (as, for instance, cyanogen, the hydrocarburets, and other very simple compounds, which cannot be obtained except from organic substances), the *practice* also. Not that the chemist can ever expect to manufacture *organized* tissues, possessed of vital endowments: these are produced by the operation of a living system upon *organizable products*. But we do not see any reason why these last should not be brought within his reach, and be as readily produced in his laboratory as they are in that of the plant or animal. We shall now briefly indicate what has been already done in this interesting line of investigation.

A compound radical has lately been discovered by Liebig, having the same elements as cyanogen, but in different proportions. To this the name *mellon* has been given, and it is composed of six carbon and four nitrogen. In the large number of proportionals which go to form one combining equivalent of this body, it bears a strong resemblance to organic compounds, of which this is usually a characteristic. Mellon acts as a compound radical in precisely the same manner as cyanogen; and its compounds are of parallel character.

A most important series of compound radicals are the hydrocarburets, or compounds of hydrogen and carbon. These serve as the bases in a great variety of organic compounds, which may be artificially made from them; but the chemist is still unable to form these radicals by the union of their elements, and, in his ulterior operations, is obliged to start from these as postulates or data, obtaining them from various sources, in all of which vitality has at some period had concern. Thus, a large number of different hydrocarburets may be obtained by the destructive distillation of coal, which, there is now no doubt, had its origin in the decomposition of resinous wood. It is a curious fact, which may be observed in passing, that in several of these hydrocarburets the relative proportions of the elements are the same, whilst the form of the body is different, its combining equivalent also varying. Thus, in *olefiant gas*, two proportionals of carbon and two of hydrogen are condensed into one volume. In *etherine*, a liquid obtained by Faraday from oil-gas that had been condensed to render it portable, four volumes of each ingredient are condensed into one. An oily liquid, described by Dumas under the name of *cetene*, contains sixteen volumes of each. And he also describes a compound (not yet obtained in a separate form), to which he gives the name of *methylene*, in which he calculates that one volume only of each occupies the same compass.

For our present purpose, however, it will be better to confine ourselves to those hydro-carburets which have been ascertained to serve as compound radicals in well-known organic combinations, that were formerly regarded as resolvable only into their ultimate constituents. The volatile oil of turpentine, in a state of great purity, consists of eight proportionals of hydrogen and ten of carbon, together forming a compound radical, to which the name *camphene* has been given. This unites with oxygen in two proportions, forming *camphor* with one, and camphoric acid with two. The volatile oil of lemons consists essentially, and almost solely, of a corresponding substance, termed *citrene*, which contains the same ingredients, in the same proportion, but in only half the absolute quantity for each combining equivalent. From the researches of Dumas it appears that most of the volatile oils containing oxygen, as those of anise, peppermint, &c., owe this ingredient to the camphor they contain, which is dissolved in variable proportion in the liquid carburets of hydrogen that essentially form them. Here is another instance in which *ultimate* analysis would have completely failed to indicate the mode in which the elements are combined.

A still more interesting compound radical was discovered, a few years since, by Liebig and Wöhler; a most unexpected relation being disclosed by the knowledge of it, between that highly poisonous substance—the oil of bitter almonds, and the inert body benzoic acid. The base of both these, termed *benzule*, consists of fourteen carbon, five hydrogen, and two oxygen. This, in union with one additional equivalent of hydrogen, forms the oil of bitter almonds; whilst, with one of oxygen, it forms benzoic acid. Hence, by merely substituting an atom of oxygen for one of hydrogen, the former compound is converted into the latter; and, accordingly, the essential oil, if exposed for some time to air, spontaneously undergoes this change, and is metamorphosed, from one of the most virulent of poisons, into a mild innocuous acid, an ingredient in many animal as well as vegetable excretions. Lowig has traced a similar class of compounds, derived from the oil of the spiræa ulmaria.

The knowledge of these facts is very important, not only in regard to the general principles of the science, but also in reference to the opinion we may form of the relations between alcohol, ether, and olefiant gas. In the time of Lavoisier, when the task of analysis was limited to the determination of the ultimate elements of a body, and of the proportions they bore one another, nothing more was attempted than to set down the amount per cent. of oxygen, hydrogen, and carbon, present in the several bodies of this class which were then known. But it has since been discovered, that a very simple and beautiful relation subsists between alcohol and olefiant gas; and that its composition may be represented by one atom of the gas and one of water. Now sulphuric ether, which is derived from alcohol by distilling it with sulphuric acid, has exactly the same constituents, but in different proportion, the amount of water being only half that contained in alcohol. Hence Dumas regarded olefiant gas as serving as the base to both these bodies, standing in the same relation to water as alkali does to an acid, and consequently readily entering into combination with it. Thus, one atom of water with two atoms of olefiant gas would constitute ether; whilst two atoms of each form alcohol. It is contended by Liebig, however, that it is more conformable to analogy to regard ether as the oxide of an unknown base, containing one atom more of hydrogen than exists in olefiant gas; and alcohol as a compound of this oxide with one atom of water. It is no argument against such a view, that this base has not been obtained in a separate form; for the same is the case with *benzule*, yet it may be transferred, like cyanogen, from one compound to another. It has been, we think, successfully shown by Liebig, that no water *as such* exists in ether; but that this substance is to be regarded as an oxide, being neutralized by acids, and forming with them a class of compounds analogous to the salts. It would appear, from a recent memoir, that Dumas has in a great degree adopted the doctrines of his opponent, to the abandonment of his own.

We have judged it advisable to abandon for a time the order of the propositions which we adopted from Dr. Daubeny's pamphlet; in order that we might bring before our readers, somewhat more fully than he has done, a view of the direction in which our knowledge of organic chemistry is now being improved and extended. It will be convenient for us now to accompany him in his remarks upon the sixth proposition, which affirms that "organic bodies have their particles held together, partly by what is called the living principle, so that the laws which operate upon inorganic matter are in many instances suspended by its influence." "This," observes Dr. Daubeny, "seems, to say the least, questionable, and the corollary founded upon it;" as to the impossibility of producing by artificial means, any of those compounds which result from the principles of life, is contradicted by fact.

"We know already of at least two, if not of more, distinct substances, proceeding ordinarily from living processes, which can be formed at pleasure by art. Such, for instance, is formic acid, which is excreted by ants, but may also be procured very readily by chemical means. Such, too, is urea; which Wöhler has succeeded in producing by the action of ammonia upon cyanogen. The crystals formed are identical with those of urea; whilst the composition is the same as that of the salt termed cyanate of ammonia, the properties of which are very different. These two, therefore, are isomeric bodies, and one of them, though it derives its existence from the ordinary processes of life, is capable of being produced without the intervention of the living principle at all.

"There is little doubt that the progress of research will bring to our knowledge many similar cases; and that it will eventually appear that all the other secretions or excretions of animals and vegetables are only so far dependent upon life, inasmuch as, in consequence of the favorable temperature which it sustains, the constant circulation of the fluids it occasions, and their exposure to external agents in vessels of different shapes and dimensions, a mechanical separation of the ingredients of the blood is effected in some instances, and a chemical change produced in its constitution by catalytic action in others." (p. 58.)

If the vital principle, Dr. Daubeny afterwards justly remarks, operates directly upon the elements of the living structure, imparting to them new affinities or suspending old ones, there would seem to be no good reason why the decomposition of the organic structure should not necessarily and immediately ensue upon the abstraction of the principle of vitality; whereas it is well known that these changes only take place when favoured by a suitable temperature, and promoted by the agency of external causes.

"The laws which regulate the decomposition of organic matters bear a sufficiently close resemblance to those that prevail amongst mineral bodies. The more complicated their structure may be, the more readily are they found to be resolvable into their component parts; for the greater the number of proximate principles which an organic substance may contain, the more the chances are multiplied, that something will interfere to disturb the balance of the sustaining affinities. The putrefaction, then, of vegetable and animal matters appears to be produced, not by any sudden cessation of those affinities which had previously bound their respective elements together, but by the predominance over them of other natural forces, which we may without much difficulty conceive to have been controlled under the circumstances in which the living body is placed; nor does there seem any sufficient reason for calling in the intervention of an occult principle to explain that, to the solution of which, by known causes, every fresh advance in chemical knowledge seems to bring us into closer approximation." (pp. 58-9.)

The following concluding remarks strike us as peculiarly apposite:

"Some, indeed, like the late Mr. Abernethy, seem to flatter themselves that they are making head against materialism, by assigning to the direct agency of a vital fluid the processes of the animal economy. But I have always felt that those who, in imitation of him, would assign to that immortal principle of our nature, which manifests itself in the operations of thought and intellect, any concerns in the functions of the perishable body, more direct and immediate than that which it may exert through the medium of the nervous system; so far from establishing on a surer foundation the doctrine of the soul's immortality, are, in fact, degrading that *divinæ particula auræ* to a level with electricity, chemical affinity, and other influences, which affect equally inanimate as well as animate matter...When we suppose that any real explanation is afforded of the phenomena, by ascribing them to the operation of the vital principle, or to any vital affinities, which is merely a less simple mode of expressing the fact, we are indulging in one of those delusive attempts to substitute words for ideas, which have so much tended to retard the progress of physiological science." (pp. 35-6.)

With these remarks we need scarcely say that we entirely concur; and we rejoice to think that such sound views are making their way amongst intelligent men. Dr. Daubeny's clear and forcible expression of them may do something, we hope, to open the eyes of those who think it necessary for the interests of religion to excite the *odium theologicum* against any one who impugns the doctrines, of which nothing but the blindness of prejudice can prevent their distinguishing the fallacy.*

We shall now follow Dr. Daubeny through his remarks (sometimes abridging and sometimes expanding them) upon the first five of the propositions above stated; having already disposed of the sixth. The *first* question we shall notice is that of the infinite divisibility of matter, which remains (Dr. Daubeny conceives) much on the same footing as that upon which it has rested for several years past. Passing by a mathematical argument, founded on the known limits to the atmosphere surrounding the sun and planets, we shall notice a statement quoted from Mr. Whewell's Bridgewater Treatise, which the researches of Ehrenberg show to require great modification. "The smallest microscropical objects which can be supposed to be organic are points or gelatinous globules, or threads, in which no distinct organs, interior or exterior, can be discovered. These, it is clear, cannot be considered as indicating an indefinite progression of animal life in a descending scale of minuteness. We can, mathematically speaking, conceive one of these animals as perfect and complicated in its structure as an elephant or an eagle, but we do not find it so in nature. It appears, on the contrary, in these objects as if we were, at a certain point of magnitude, reaching the boundaries of the animal world. We need not here consider the

* See Edinb. Med. and Surg. Journal, Jan. 1840.

hypotheses or opinions to which these ambiguous objects have given rise; but, without any theory, they tend to show that the subordination of organic life is finite on the side of the little as well as of the great." Now, we are not going to enter upon a discussion on the validity of Ehrenberg's statements respecting the complexity of organization in polygastric animalcules, but we think that it will be interesting to our readers if we present them with some of his recent estimates of the degree of minuteness of the particles of which the microscope can take cognizance.

It may be observed, *in limine*, that every improvement in the powers of the microscope has brought into view whole groups of living beings, whose existence was not even suspected, and has laid open a complex structure in others which were previously considered to be of the simplest possible character. We have no doubt that the hints thrown out by Ehrenberg, in his Essay on Organic Molecules and Atoms,* written about five years since, have been subsequently realized. Of this paper we shall now give a brief summary:

"I could plainly distinguish (says Ehrenberg), with a microscope magnifying 800 times, *monads* which had been filled with colouring nutritive substances, and which possessed voluntary motions, but the entire and greatest diameter of whose body only amounted to $\frac{1}{1500}$ or $\frac{1}{2000}$ of a Parisian line. To this smallest animal form yet discovered I have given the name of *monastermo*. I could perceive in the largest individuals as many as six, and in the smallest as many as four internal sacs, coloured blue by indigo, which at times did not occupy quite half of the dimensions of the animal. Such a sac, therefore, of the *monastermo* must be at the most $\frac{1}{12000}$ of a line in diameter. At the upper part of this animal is seen, as in all the monads, a powerful movement of particles still smaller than themselves when these have approached them; and it is therefore probable that it has a fringe of from ten to twenty cilia at the mouth-aperture, like that which can be distinguished in the larger monads. Further, even if we do not suppose the single colouring particles with which the stomachs are gradually filled to be very numerous, we must admit, from the roundness of the sacs, that there must be at least three particles in each. This affords us proof of the existence of material colouring particles of red and blue, moving freely in the water, which measure $\frac{1}{36000}$ part of a line, or $\frac{1}{432000}$ part of an inch in diameter. If we found our computation on the size of the smaller monads these particles would not exceed $\frac{1}{576000}$ of an inch in diameter. If the cilia really exist, they must probably have a less diameter than $\frac{1}{38400}$ of a line, or they would be themselves visible with the highest powers of the microscope. [Ehrenberg subsequently states it as the result of his experiments on the limits of microscopic vision, that squares of $\frac{1}{48000}$ of a line each way can be distinguished by a careful observer with a clear magnifying power of 1,000.] Further, in the larger infusoria of similar structure it is seen that, when the coloured globules contained in two digestive sacs appear to touch one another, they are separated by a membranous partition, bearing but a small proportion to the diameter of the sac: say, one-twentieth at most. This would amount to $\frac{1}{320000}$ of a line, or $\frac{1}{3840000}$ of an inch in monads $\frac{1}{20000}$ of a line in diameter. The proportion which the granules constituting the ova of larger animalcules bear to the size of the parents is generally from $\frac{1}{40}$ to $\frac{1}{80}$ of their diameter. We may then consider the diameter of young monads, just come from the egg, to be almost $\frac{1}{60000}$ of a line, and perhaps much less. These would have digestive sacs, whose diameter in the same proportion would be $\frac{1}{480000}$ of a line; and the thickness of their walls would not exceed $\frac{1}{9600000}$ of a line, or $\frac{1}{115200000}$ of an inch. It is by no means certain that the *monastermo* is the smallest species existing; for, under favorable circumstances,

* Taylor's Scientific Memoirs, vol. i. p. 568, et seq.

wandering shadows of much smaller monads have been observed, which some future improvements in the microscope may enable us to study with the same precision."

These calculations are made upon fair data, and are not to be regarded as conjectural.

"They plainly demonstrate," says Ehrenberg, "an unfathomableness of organic life in the direction of the smallest conceivable space; and if the word *infinity* be too much for what we know at present, let the word *unfathomableness*, which I have purposely employed, avert from me the reproach of exaggeration, and establish the points of view under which the physical, chemical, and physiological enquiries of our days, should they be rendered fruitful by new powers, will have to take, and what deviations they have to avoid." This, we think, is the correct mode of viewing the subject. We do not see how the *infinite* divisibility of matter can be ever proved, for all man's operations are finite. But that no human means will ever meet with a limit, we are fully persuaded.* On this, as on many other subjects, we deem Mr. Whewell's conclusions extremely hasty. We shall now return to the purely chemical view of the subject, for which we shall have recourse to Dr. Daubeny:

"Although the abstract doctrine with respect to the existence of ultimate atoms seems still to maintain that ascendancy over the opposite opinion, which it acquired from the period that Dalton first established the laws of definite proportions, it must be admitted at the same time that combinations amongst bodies may be more readily explained by imagining them to take place between certain definite groups of these atoms, than by assuming, as the father of the atomic theory preferred to do, that they resulted from the union of simple atoms of each ingredient. Thus much, at least, appears clear, namely, that when a body is resolved into the state of gas, so that its several parts exert a mutual repulsion, the parts so repelling each other are not single atoms, but groups of them." (p. 14.)

This supposition is necessary to account for the fact that, in some instances, when two gases unite to form a third, the total bulk is not diminished, as it would be if the atoms, between which that repulsion operates which keeps them in the state of gas, were the same with those which chemically unite; and which, therefore, are diminished in number to one half. It also affords a satisfactory explanation of the want of correspondence between the combining weights and combining volumes of certain gases, as well as of the simple numerical relation always subsisting between these. Dumas has hence proposed to designate that description of molecular groups, which constitute the extreme division of which matter is susceptible by heat, by the term *physical atoms;* and those simpler groups, into which their affinities for other bodies often subdivide them, *chemical atoms.* From the experiments of MM. Dulong and Petit on the relation between the specific heat of various bodies and their atomic weights, it is inferred by M. Dumas, that the chemical atoms, or smallest combining proportionals, are not themselves the ultimate particles of the body, but contain these in a number varying with each element.

* Muncke (Handbuch der Naturlehre, Heidelberg, 1829,) demonstrates that a grain of indigo may be divided into thirty-eight *billions* of visible parts: and when we consider that a human being could not count a single billion, working night and day, in a shorter period than *thirty thousand years,* we may form a faint conception of the number of particles in a grain of matter.

The following may be regarded, in Dr. Daubeny's opinion, as expressing the present state of knowledge on this subject:

"On the mathematical question which stands on the threshold of the subject, it cannot be expected that much new light should have been thrown. We are still, therefore, equally at liberty to embrace the theoretical doctrine of the capacity for infinite division necessarily inherent in matter, or the more metaphysical and refined hypothesis of Boscovich, who deduces the primary qualities of all natural bodies from the existence of a number of ultimate points, destitute of all properties save that of mutual attraction and repulsion, operating at certain distances, and obedient to certain laws.

"But the present stage of experimental knowledge, whilst it leaves us at liberty to speculate as before with respect to the inherent capacity of division, which may belong to the smallest conceivable portion of matter, as well as to the largest, affords at the same time grounds for the belief, that the Author of Nature has placed somewhere in the scale of minuteness a point beyond which no natural force can carry division. Now a body, of whatever dimensions we may assume it to be, which is held together by a force superior to any which can ever be brought to divide it, we denominate an *atom*.

"The relative weight of these atoms may probably be indicated by the specific heats assigned to the several substances.

"With these atoms, however, chemistry, strictly speaking, has no concern; but it is with groups or assemblages of them, held together by a certain cohesive force, which is proof against every other sort of attraction, that this science is conversant. These assemblages of atoms (the *chemical atoms* of M. Dumas), uniting with each other in various proportions, produce combinations according to the law of definite proportions, and are mutually displaced by the operation of chemical affinities.

"Lastly, by converting a body into gas or vapour, we separate it into other groups of particles (the *physical atoms* of M. Dumas), consisting of one or more of those between which chemical union takes place." (pp. 17-20.)

The first part of the *second* proposition—which implies that the chemical properties of each body, so long as it remains uncombined, are, like its physical ones, essential and constant—requires great modification in consequence of the important discoveries of Faraday and others, as to the relation between *electrical attraction* and *chemical affinity*. Whether or not we regard these powers as coincident, it is unquestionable that the latter is greatly influenced by the former, so that a body is often affected very differently by the same reagent, according to the electrical condition that has been induced in it. Some very curious facts bearing upon this subject have been recently brought to light by Professor Schœnbein of Basle. These, from their general tenor, might lead us at first sight to believe that the affinity of iron for nitric acid, instead of being inherent in the metal under all circumstances, was superadded to it by certain extraneous influences, its existence being dependent upon the relations of the metal at the time to electricity, or on some other causes equally obscure.

"1. If an iron rod be raised at one extremity only to a red heat, it will not be acted on by nitric acid of the sp. gr. of 1·35. 2. This immunity from the action of the acid may even be imparted to a second rod if brought into connexion with the first. Thus, if the heated wire be made to touch a second, and both be plunged into the same acid, neither one will be acted on. The same immunity is obtained if an iron wire, plunged into nitric acid, is simply made to touch a wire of platina. 3. The same wire, if made the positive electrode of the galvanic battery, is not acted upon by the acid, though it transmits the galvanic current, and consequently decomposes the water present; whilst, on the other hand, it is vehemently attacked by the same acid when in connexion with the negative electrode. 4. The same immunity is conferred on the metal, by immersing it for a few moments in acid, after which the action entirely ceases; and it may then be sometimes renewed by various mechanical methods, as by rubbing it with a copper wire, with glass, or in other ways." (p.21-2.)

Modern discovery, then, compels us to recognize a distinction between the physical properties of matter and their chemical ones, inasmuch as the former are always essential and inherent, the latter sometimes induced by extraneous agencies. But there is nothing to contradict the belief, that the conditions, on which depend the capacity of being affected in this manner, are in themselves as permanent, and subject to laws as fixed and definite, as those which seem more directly to belong to their constitution and nature. But, if the chemical properties of matter are affected by changes in their electrical condition, it is not unreasonable to suppose that a different arrangement of the particles of the same species of matter may alone be sufficient to bring about a change in its chemical constitution, by altering its relations to electricity. Hence we are prepared, on theoretical grounds, for receiving the new doctrine which has so much changed the face of chemical science, and on which we have already expatiated, namely, that many substances which are known to be of a compound nature, are nevertheless in some sense to be regarded in the light of elements.

The doctrine of *compound radicals* has lately been extended, as we have already hinted, to inorganic chemistry. These views were, we believe, first propounded by Liebeg; and he is supported in them by Professor Graham, who may justly rank, we think, as the highest British authority on philosophical chemistry. Formerly we were in the habit of assuming that four or five combinations might take place between one elementary body and another; thus, that sulphur with one atom of oxygen formed hyposulphurous acid; with two, sulphurous acid; and with three, sulphuric acid. But, according to these chemists, the hyposulphurous acid unites as a compound radical with an additional atom of oxygen to form sulphurous acid; and sulphurous acid, in like manner, acts as a compound radical, forming sulphuric acid with another atom of oxygen. Again, when sulphuric acid unites with soda, it is believed that the acid takes the oxygen of the alkali to form a fourth compound, to which the name *sulphatoxygen* has been given, which unites as a compound radical (performing the part of an elementary substance) with the metallic sodium.

Nor is this the only change which it is proposed to introduce into the mode of considering these acids. According to the old notions, it was necessary to suppose that some of these acids derived their acidity from oxygen, others from hydrogen, others from chlorine, iodine, &c. In order to remove this anomaly, it was originally suggested by Davy, that what we call the oxygen acids are, in fact, combinations of hydrogen with a compound base containing a certain number of atoms of oxygen. Thus hydrous sulphuric acid is usually represented as sulphuric acid + water. Whereas it is possible to consider it in the light of *sulphatoxygen* and hydrogen; hydrogen being the acidifying principle here, as in the case of muriatic acid. This view has been fully developed by Professor Graham in his Elements (p. 160); and, more recently still, Liebig has given it the weight of his name and authority. It is supported by the fact, that the power of saturation which an acid possesses is dependent upon the amount of hydrogen present in it; and, moreover, that every known substance which exhibits acid properties contains hydrogen, either by itself or in the form of water. For, when destitute of this principle, as is the case with the anhydrous sulphuric, phosphoric acids, &c., it exerts no chemical reaction whatsoever. Still we *know* nothing of the existence of such a compound radical as *sulphatoxygen;* and the chemist who assumes it may be charged with having fallen into the same error with his predecessors, who imagined the hypothetical principle phlogiston.

It will be evident, however, to any one who takes the trouble to examine the subject for himself, that the doctrine of *compound radicals*—that is, of combinations of two or more elements, which, in their chemical relations with other bodies, correspond with simple substances—enables us to bring organic compounds under the same general laws as those of the mineral world; and that the extension of it to inorganic che mistry will, in the end, do much to simplify the principles of the science.

"We perceive, then," concludes Dr. Daubeny, in reference to the second proposition, "that it cannot be adopted without considerable modifications; for neither, on the one hand, do those substances which we still hold to be elementary manifest relations to others at all times the same in degree or intensity, nor, on the other, are those which constitute the basis of organic substances, and which stand on the same footing with respect to the rest as the elements of mineral bodies do to the salts and acids to which they give rise, necessarily themselves simple, or composed of one kind of matter. They resemble, indeed, rather the elements represented by Ovid and other writers of antiquity, the bases of other bodies, but yet capable themselves of conversion from one shape into another." (p. 35.)

The *third* proposition, which lays down that compound bodies, formed by the same elements in the same proportions, must have the same properties, and that a difference in the ingredients must produce a difference in the properties of the compound, must also undergo considerable modification, in consequence of the discoveries of recent times. It has been found, for example, that the same ingredients, united together in precisely the same proportions, will often give rise to several compounds as distinct in their nature as those whose component parts themselves vary. Such bodies are now distinguished by the term *isomeric;* denoting that they consist of equal parts or proportions of the same elements. In some instances, the distinction between them appears to arise from the different degrees of condensation which the ingredients have undergone; as in the case of the hydrocarburets already alluded to. In another class, the difference between two isomeric bodies may be traced to a difference in the processes by which they have originated. But there are many to which no such explanations apply. In all these instances there can be no doubt a different arrangement of the component particles has taken place in the two resulting bodies; and this accounts for the fact of their assuming different crystalline forms, and having different relations to light, electricity, and other physical agents, and also for their combining proportions not corresponding. But why the same ingredients should affect one rather than the other arrangement is a question still veiled in obscurity.

Nor is the assumption of different properties by bodies which, in their constitution, appear to agree, the only point on which recent discoveries clash with the established principles of the chemistry of a former era. The same substance will sometimes assume two or more crystalline forms, without any alteration of its qualities supervening. Bodies which thus present themselves under two different primary forms are called *dimorphous;* and the number of these already known is so great, that it may perhaps be doubted whether there be a body in nature which constantly occurs in the same physical condition under all circumstances. If we regard *isomerism* as resulting from a different arrangement of the *chemical* atoms, we may perhaps attribute *dimorphism* to a variety in the arrangement of the *physical* atoms; or, to use an analogy employed in a corresponding case by Lucretius, the first class of bodies may be compared to different words formed by the transposition of the same letters, whilst, in the second class, the arrangement of the syllables only is reversed, the letters in each syllable remaining the same. As to the cause of the variety of crystalline form assumed by the same body, we are still much in the dark. The researches of Mitscherlich, however, have shed some little light on the question. He has shown that heat, in many cases, tends to modify the crystallization of a body, by causing it to expand unequally in different directions, enlarging the acute angles, and thus imparting to it a tendency towards the cube. Hence the same substance, if made to crystallize at a high temperature, may assume a different form from that which it would take at a lower one; and this is actually found to be the case with carbonate of lime, which, when deposited from a solution in the cold, assumes the rhomboidal form of calcerous spar, but when from water of a higher temperature becomes arragonite.

If it be true that a mere variation in the arrangement of the same particles is alone sufficient to produce an entirely different material, we are naturally led to speculate as to the possibility that the substances which, inasmuch as they have hitherto resisted our powers of decomposition, we regard as simple, may themselves be isomeric bodies, or be produced by a different arrangement of the same elementary matters. Thus have the beautiful discoveries of Mitscherlich and others brought us back to the very speculations which engrossed the alchemists of old; so that it has happened in this case, as in that of the atomic theory itself, that modern science has incidentally lent support to views which had been originally brought forward on grounds altogether different, and by a class of persons whose habits and principles of reasoning were as opposite as possible from their own.

We are rather surprised that Dr. Daubeny has not referred to the phenomena which may be regarded as the converse of these two, namely, *isomorphism.* This term is employed to express the fact, that, in many instances, one element of a combination may be withdrawn and replaced by another without any change in the external form of the crystal, or in its general physical properties. The bodies which may thus be substituted for one another form groups, which are said to be *isomorphous.* One of the most remarkable of these contains the acids formed by the union with arsenic and phosphorus with oxygen, which have altogether a most remarkable correspondence—arsenious acid having a strong resemblance in all its chemical relations to phosphorous, and arsenic to phosphoric. Another group contains the sulphuric, selenic, chromic, and manganic acids, which may be substituted for one another without the form of the crystal being changed. In another group are included the sesquioxides of aluminium, iron, chromium, and manganese. Several other groups might be enumerated, did space permit. It is, of course, necessary that the combining proportion of the substituted element should be the same as that of the one withdrawn. The variety which may be produced in the constitution of alum affords an interesting example of these properties. The alumina may be replaced by the sesquioxides of iron, chromium, or manganese: ammonia may be substituted for the potash, and selenic, chromic, or manganic acids for the sulphuric; so that the composition of this salt may be partly or entirely changed, without its external form being affected. Isomorphous substances have often very close points of resemblance, independent of external form. Thus the vapours of arsenic and phosphorus have nearly the same odour: many of their precipitates correspond in colour; they both form gaseous compounds with hydrogen; they differ from nearly all other bodies in their mode of combining with oxygen, but agree with one another; and their salts are disposed to combine with the same quantity of water of crystallization. A similar analogy exists between sulphur and selenium, baryta and strontia, lime and magnesia, cobalt and nickel.

It is evident that isomorphism affords additional weight to the speculations just now hazarded in reference to the compound nature of many substances now regarded as elementary. They can *only* be at present regarded, however, as speculations; but we think the day is not very far distant when their truth or falsity will be determined.

The *fourth* proposition—in which it was stated that "decomposition is caused by the approach of a foreign substance, possessing a stronger affinity for one of the ingredients of a compound than that which binds them mutually together, so that in every such case the decomposing body may be expected to enter into union with one or other of the elements of the compound it destroys"—cannot, in the present state of our knowledge, be received without considerable limitation, although it might formerly be regarded as an obvious consequence of the recognized principles of chemical combination. It has been found that there are substances capable of causing decompositions, and of bringing about new unions between certain elementary and compound bodies, merely by virtue of their *presence*, and without themselves combining with either constituent. The numerous cases in which this is assumed to take place have led to the invention of a particular term, *catalysis* (dissolution), to indicate the property in question; thus marking the distinction between what takes place here and that which occurs in a process of *analysis*, where a new substance being presented to an existing combination annuls it by uniting itself with one of the constituents. The following are instances of the kind.

Platinum, especially when in that state of minute division in which it is known as spongy platinum, has the power of occasioning the union of various inflammable gases with oxygen, at a temperature at which they would otherwise remain together without combining. The peroxide of hydrogen, or oxygenized water, is resolved into water and oxygen, (the latter passing off with effervescence), by the contact of various substances, such as the alkalies, metallic oxides, as well as metals, which do not combine with the oxygen liberated. In like manner, the nitro-sulphuric acid of Pelouze is decomposed, by spongy platinum, by oxide of silver, by metallic silver, by powdered charcoal, and by other substances, which are not acted upon by any of the constituents of the compound which they decompose.

These cases of catalytic action are taken from the inorganic kingdom; but they occur much more commonly in the processes of organic chemistry. Of the vegetable principles upon which it is distinctly exhibited, one of the best instances, perhaps, is starch; which undergoes the changes into sugar, gum, and the substance called by the French chemists *dextrine*, under the operation of agents which do not enter into union either with it, or with the ultimate principles of which it consists. This change is effected in the living vegetable by the operation of a secretion termed *diastase;* but the chemist can imitate it by various other substances. The action of sulphuric acid upon alcohol, in producing ether, appears to be of the same nature. It has commonly been attributed to the affinity of the acid for water, which causes it to abstract from the alcohol the proportional which it contains, and so to convert it into ether; but it has been objected that potass, chloride of sodium, quicklime, and other substances which possess an equal affinity for this liquid fail of converting alcohol into ether; and Mitscherlich has shown that, if alcohol be poured upon sulphuric acid at a temperature higher than that of boiling water, a proportion of water and ether, exactly equivalent to the alcohol employed, will distil over; so that the sulphuric acid, having united with neither of the constituents, must be regarded as having acted catalytically. This new principle furnishes us with a clue to the formation of those many products, which result from the selfsame fluid in the living animal and vegetable. In this way we may comprehend how from the sap alone, sugar should be produced in one part of the plant, camphor in another, an essential oil in a third, caoutchouc or milky juices in a fourth; and how, from the blood of the animal, the various secretions and organizable products should be elaborated, which are characteristic of the different parts.

The proposition under consideration will also, in another sense, be found to embrace only a portion of the truths which modern chemistry has revealed to us; since it overlooks the very remarkable influence which cohesive attraction exerts upon chemical combinations. This is remarkably exemplified in the solubility of recently precipitated silica in water, as compared to its entire insolubility when in a state of aggregation; and the knowledge of this fact affords us the only clue we possess to the knowledge of the mode in which this is introduced into the juices of plants, and other positions from which its nature would seem to exclude it. But, according to Dr. Daubeny, something different from what is commonly known as cohesive attraction must be here understood.

"There is another mode (he remarks) in which the attraction between like bodies appears to operate, bearing, as it would seem, somewhat of the same relation to the cohesive attraction which binds together their particles, as the attraction between opposite masses of matter of dissimilar natures, when in opposite electrical conditions, bears to the elective attraction which exists between the ultimate atoms of each. This has frequently been applied by geologists and others to explain certain phenomena that have presented themselves to their observation; but has not, so far as I am aware, received the attention it deserves from chemists. It is well known that, if an intimate mixture of finely comminuted sand and clay is left together, as in the common operations of pottery, the silica will, after a certain time, be found collected in clusters. Dr. Faraday has shown that the same principle causes camphor, if suffered to evaporate in a glass vessel, to arrange itself round certain nuclei on the surface, instead of being equally disseminated throughout. The same principle has been called in to explain the nodules or concretions of one kind of stone, as of flint, so frequently found to occur in rocks of another description. The adhesion of smooth plates of glass or of metal, when brought into close contact, and the property of water to adhere to solid substances, which gives rise to the phenomenon of capillary attraction, and to the hygrometric power belonging to certain earths, may be accounted for in the same manner; as may also the capacity, which most porous substances possess, of absorbing, and even condensing within their pores, the gases with which they happen to be surrounded. The curious manner in which gases appear in some cases to adhere to metallic surfaces, without actually combining with them, seems another fact in illustration of the same principle.

"I conceive that the circumstances by which this singular kind of attraction is regulated deserve a more full investigation, and that it is highly proper to distinguish it by a separate name from the cohesive attraction subsisting between the particles of matter. Perhaps the term *adhesive* attraction, which has been already proposed for it, would be sufficiently characteristic. It has been questioned, indeed, whether this species of attraction is essentially different from that which we denominate cohesive; and it may safely be conceded that the primary cause of both will most probably turn out to be the same. But so, indeed, may cohesive attraction itself, according to the ingenious generalization of Mossoti, be included under the same law as that of gravitation; and the chemical affinities of bodies be regarded as the consequences of their physical properties, as Persoz has attempted to demonstrate.

"None of these hypotheses however, even if they were fully established, and more completely developed than they can claim to be, ought to prevent us from ranging under distinct heads, and treating as separate branches of science, these several phenomena, regarding the attraction which operates between masses of matter distinct from that which takes place between their particles, as electrical attraction is from chemical affinity; or as the repulsive energy exerted by heat upon masses of matter is from the same force which imparts an elastic condition to their particles." (pp. 51-4.)

The only one of the propositions first stated that now remains for us to consider, is that one which set forth "that the water attached to many bodies, though essential to their crystallization, does not in other respects affect their properties." This has been greatly modified by recent researches, especially those of Professor Graham; which have shown that water contributes, not merely (as was supposed) to the particular geometrical form which a mineral has a tendency to assume, but likewise to the chemical relations it possesses towards other substances. Professor Graham establishes, in the first place, that acids, such as the phosphoric, combine with water in certain definite proportions, just as they do with bases; that one atom of water is in most cases necessary to their existence; but that they disengage this atom when they unite to an equivalent quantity of any other base. Thus water acts as an acid with bases, as in the case of hydrate of potass, lime, &c.; and as a base in the instance of acids, as the liquid sulphuric and nitric acids testify.

We may, therefore, distinguish several states in which water exists as the constituent of a compound. 1st. As *water of crystallization;* that is, essential to the crystalline form which the substance assumes. 2d. As an *acid*, combined with alkalies, earths, &c. 3d. As a *base*, being essential to the existence of acids, with which it is combined. To these Professor Graham has since added a fourth condition, in which it may occur; forming what in his last Memoir (1837) he has called *constitutional water*. In this case, we have a certain proportion of water united to a given salt, but capable of being driven off from it by another, in consequence of the stronger attraction which the latter exerts. Thus, the sulphates of magnesia, zinc, &c. contain besides their water of crystallization, a proportion of constitutional water, which may be replaced by sulphate of potass. This constitutional water is expelled with more difficulty than water of crystallization. Thus, of the seven equivalents of water which crystallized sulphate of zinc contains, six may be easily driven off, but one remains in combination at 410° and all inferior temperatures; and it is this one which is replaced by sulphate of potassa, so as to form what has been hitherto termed a double salt, without any change in external form. It may, perhaps, be questioned whether this constitutional water is not really acting as a base or acid; the salt with which it is united serving as a compound radical. The curious analogy between the effects of heat on plaster of Paris and the arsenic and phosphoric acids would seem to support this view. Plaster of Paris appears to consist of a sulphate of lime and two equivalents of constitutional water. At a temperature not exceeding 270°, this salt loses its water, but retains the power of recombining with it, or *setting;* but if heated above 300°, it becomes properly sulphate of lime, and loses the tendency to recombine with water. Now, according to the researches of Professor Graham, phosphoric acid cannot be obtained except in combination with *three* equivalents of water, which is here termed *basic*, and which may be wholly or partly replaced by other salifiable bases. Thus, there are three phosphates of soda, known under the names of the *alkaline*, *neutral*, and *acid* phosphates; in which *one*, *two*, or *three* of the atoms of water have been replaced respectively by the soda. But on exposing phosphoric acid to heat, one atom of water is driven off; and an acid remains, termed the paraphosphoric, having the same proportions of phosphorus and oxygen as exist in phosphoric acid, but combined with only two equivalents of water, and capable of only receiving *two* proportionals of a base in their stead. And if this acid be exposed to a still higher temperature, another atom of water is driven off, and the product is metaphosphoric acid, which is only capable of uniting with one proportional of a salifiable base. Arsenic and telluric acids have a tendency to form similar combinations.

This principle, that the acid possesses an affinity for just so many atoms of a base as the number of atoms of water with which it is already combined, is probably by no means limited to the cases just mentioned; and it has been ingeniously brought, by Liebig, under the general fact, that no acid has any tendency to combine with a base, except when it already contains water, or at least hydrogen, in the following manner.

Phosphoric acid owes its acid properties to the presence of a certain proportion of water; and without it, like dry tartaric acid, has no tendency to combine with bases. Accordingly, when one atom of water is abstracted from phosphoric acid, the paraphosphoric acid which remains may be regarded as an admixture of one proportional of anhydrous (and therefore inactive) phosphoric acid, with two which still retain their combining power. And when two atoms are abstracted, so as to form metaphosphoric acid, we may view this as really composed of two proportionals of anhydrous, and one proportional of active, phosphoric acid.

The important part which, in these cases, water seems to perform, in imparting to the compound into which it enters as an ingredient its most active properties, and the impossibility of pointing out a single acid destitute at once of water and of its constituent hydrogen, are the circumstances that principally lend a foundation to the bold theory already noticed, which supposes all the acids to consist of a compound radical, united with hydrogen as an acidifying principle. The arguments in favour of this view are stated with much clearness by Professor Graham, (Elements, p. 389, et seq.;) but they need not be here dwelt on; as the several theories suggested on these points are admitted by the best authorities to be so nearly balanced, that new discoveries may at any moment give the preponderance to one over the other.

The only other important modification in the hitherto-recognized principles of chemical philosophy that we shall now notice is that which has been termed by Dumas (by whom it has been proposed) the *law of substitutions.* We have not been able to find any single statement of it which gives to our minds a sufficiently clear view of its nature, as distinguished from the general law of definite proportions on the one hand, and the doctrine of isomorphism on the other. It is founded upon the idea that the several elementary bodies may be divided into groups, the members of which may severally replace or be substituted for each other, in any compound into which one of them enters, without producing any essential change in its general chemical relations. This principle was first applied to the action of chlorine on organic compounds. When chlorine acts upon these bodies, for every atom of hydrogen that is abstracted in the form of hydrochloric acid, an atom of chlorine is left in its place. The same doctrine has been successfully applied to the action of oxygen and other agents upon the same bodies. Thus, in the oxidation of alcohol during the acetous fermentation, hydrogen is withdrawn in the form of water, by combining with oxygen, and at the same time the hydrogen is replaced by an exactly equivalent quantity of oxygen. By the pursuit of this principle, chemists have been led to the discovery of many new and important combinations, and their operations have been much simplified, so that they are now able to perform in a short time what was formerly the labour of years. This results from the power which is obtained by it, of predicting (in cases entirely unknown) what will be the results of particular decompositions. It seems to be one of those subordinate laws, "limiting the generality of the law of definite proportions in particular cases, diminishing the number of combinations abstractedly possible, and restraining the indiscriminate mixture of elements," which we formerly spoke of (Vol. V. p. 323,) as remaining to be discovered. One of the examples adduced by M. Dumas will perhaps set this point in a clearer light. If acetic acid be subjected to the influence of chlorine, a new compound will be formed, in which the oxygen of the original acid has been replaced by chlorine. This is called chloroacetic acid; and as it fully retains its power of saturation with a base, it must be regarded as belonging to the same *type* with acetic acid. Now if chloroacetic acid be boiled with an alkali, it is destroyed, and is changed into carbonic acid and chloroform, (a compound of six chlorine, two hydrogen, and four carbon). If acetic acid is treated in a similar manner, it may be surmised that it would resolve itself into carbonic acid, and a carburet of hydrogen; but *which* of the several hydrocarburets nearly similar in composition would be produced by it could not be predicted without the aid of this law. The knowledge that the two compounds in question are of the same chemical type, however, would enable the chemist to assert that out of at least four possible products, that one would be evolved which corresponds most nearly in composition with chloroform, namely, the light carburet, or marsh gas; this consists of eight hydrogen and four carbon, and is changed by the action of chlorine into chloroform (six proportionals of chlorine being substituted for the same number of equivalents of hydrogen), thus bearing the same relation to it as acetic to chloroacetic acid. This is what is found experimentally to be the case. Those of our readers who wish to have M. Dumas's own account of his discovery may consult with advantage his recent Memoir upon the Law of Substitutions, and the Theory of Chemical Types, (Comptes Rendus, Feb. 5, 1840; and Philosophical Magazine, May, June, and July, 1840.)

We have already recorded our opinion that the place at present held by chemistry in the curriculum of medical study is much too high; and we doubt not that it may have occurred to many of our readers, during their perusal of the foregoing pages, that, however interesting in themselves, the statements there put forth had no very direct bearing upon the objects to which the life of the practitioner is devoted. His therapeutic employment of ether is not affected by the dispute, whether it is to be regarded as an oxide of ethule or a hydrate of olefiant gas; and he can give a dose of sulphate of magnesia just as well whether he believe it to consist of sulphuric acid and magnesia, or of sulphatoxygen and magnesium. The practical applications of chemistry will be, for the most part at least, the same, whatever theory we may adopt of the abstract nature of the changes themselves. To the medical student (as we formerly observed) no more acquaintance with chemistry is essential than that which will prevent him from mixing incompatibles in his prescriptions, and will enable him to conduct, with tolerable skill, the search for the commonest poisons. To endeavour to cram him with more is, in our view, only to prevent the acquisition of, or to drive out if already acquired, knowledge of a much more useful description. We are ready to admit that the pharmaceutist ought to be further instructed; yet even here, a very moderate knowledge of chemistry will ordinarily be sufficient for those who have no ambition of exploring new fields of research, but are content to go on in the beaten path. He who devotes himself to the business of discovery in *any* branch of science must undergo a special preparation for it; and it can scarcely be fair that such preparation shall be obligatory upon every one, however limited the sphere of operation which he contemplates.

These remarks are intended peculiarly to apply, however, to the present state of chemistry. As the practitioner of medicine and the pharmaceutist have gradually been separated, chemistry, like botany, evidently belongs to the latter; so far, at least, as these sciences are limited to the supply of therapeutic agents. And the peculiar complexity which necessarily attends the present transition state of chemical philosophy, and the unsettled condition of what have been for long regarded as its most stable foundations, furnish other reasons why the medical student should be perplexed with it as little as possible. We look to a future, however, in which the discoveries now in progress regarding the real nature of organic compounds shall be brought to bear effectually upon therapeutics; and shall not only furnish us with many new and important medicinal agents, but give valuable assistance in the discovery of principles, far more certain than any we at present possess, which shall be the guide in their application. But the prosecution of the researches by which this shall be accomplished is not to be looked for from the medical practitioner. With him it will rest to bring into application the results, as they are respectively attained; but organic chemistry is a science which, to pursue with any prospect of success, requires the devotement of the whole time and attention. As we have formerly stated, we consider it an important object in general education, to communicate as comprehensive a knowledge as possible of the *principles* of science; since, by this preparation, the subsequent pursuit of any one of them will be greatly facilitated. And we are by no means disposed to exempt chemistry, even though its present condition appears so unsettled. The general expressions of its phenomena may be communicated, without the theoretical explanations of them; in the former all agree, whilst on the latter almost every teacher would give a different opinion. There is, in fact, no more essential connexion between the theory and the phenomena to which it applies, than between the question as to the abstract nature of light and the laws of optics. The latter may be taught, as every one knows, without in the least degree involving the former.

Now it is in this manner, we think, that chemistry ought to be taught at present. Those general laws should be stated which are but comprehensive expressions of facts, which remain unchanged by any modification of theory, and which serve as the guide in predicting the effects of the operations to be performed; but very little further would we go. A student who learns a particular theoretical explanation from a teacher whose authority and talents he respects, is very apt to retain it in spite of clear evidence to the contrary, even after his master himself may have abandoned it; and such a tendency must check that spirit of enquiry, which is one of the most valuable results the student can obtain from this kind of training, as well as produce positive injury by habituating the mind to the retention of what has been proved to be erroneous. There is, perhaps, more difficulty in teaching chemistry on this plan than most other branches of science, since the nomenclature is so much involved in its theory; but we are so far conservative in our tendencies, that we would rather have a public teacher hold by the old as long as he can (provided he make it clearly understood that it may not improbably have to undergo modification), than be carried about by every wind of doctrine, and be always grasping at novelty. The difficulty is no small one; especially when the teacher is himself labouring to advance the theoretical department of chemistry (as is the case with Professor Graham), and when the extent of his course encourages him to enter somewhat at large into the subject. But the mode of instruction ought to be varied according to the object with which the knowledge is communicated ; and, if all are alike instructed in the *practical principles* of the science, the medical student may afterwards direct his special attention to the details with which *he* ought to be conversant, whilst he who intends devoting himself to the higher departments of the science may enter at large upon the study of chemical philosophy.

If this be the right view of the subject, it appears that the work which is abstractedly the best on the science is not therefore the best for the use of the medical student. We regard the (modestly denominated) Elements of Professor Graham as worthy to rank with any systematic work yet published, in regard to the profoundness and comprehensiveness of its views, and the clearness with which these are stated. But we fear it is much above the grasp of those for whose use it was intended, except when expounded by himself. To those, however, who are prepared by a general knowledge of the subject to enter upon the consideration of the new views here set forth, and who are desirous of keeping their knowledge of *principles* up to the level of the times, we can unreservedly recommend the work of one who is deservedly ranked as the most philosophic of British chemists, and who has done more than any other to redeem the stain under which England lay, not many years since, of inertness and even neglect.

To the medical student, on the other hand—especially to him who has not acquired any great amount of preparatory information—we strongly recommend the work of Professor Daniell. The plan of the work originated in the author's desire to present to students in chemistry an elementary view of the discoveries of Dr. Faraday in electrical science. "From the very first publication of his *Experimental Researches in Electricity*," says Mr. Daniell, "I have felt that from them chemical philosophy will date one of its most splendid epochs ; and perceiving, at the same time, that the results bear upon them the great impress of natural truths—namely, that they simplify while they extend our views—I have from the first availed myself of them in my instruction to my classes." Upon considering the best mode of carrying his designs into execution, he became convinced that these doctrines could not be simply and intelligibly stated, without a preliminary notice, embodying an account of other laws of matter. "Thus, I was gradually led to include in my plan such a preparatory view of the forces which may be said to concur to the production of chemical phenomena, as it is absolutely necessary for the student to master, who aspires to anything beyond the mere manual operations of practical chemistry." In accordance with this plan the author, after some general remarks of great excellence on inductive philosophy, considers what is meant by *matter* and *force ;* the varieties of force ; the laws of gravitation, with specific gravities ; elasticity, elastic fluids and sound ; homogeneous attraction and repulsion ; heterogeneous adhesion ; and crystallization. He then proceeds to the laws of heat and light, and thence to electricity and magnetism. Thus prepared, he enters upon what are usually termed chemical phenomena ; and, after a general view of the nature of chemical affinity and the law of definite proportions, he illustrates it by a sketch of the properties of the non-metallic elements and their most important compounds. From this he passes to the more complex subject of concurring forces, and the variation in the phenomena produced by the influence of these; and the remainder of the work is devoted to an exposition of the connexion between electrical and chemical phenomena, as developed by the researches of Faraday. We have seldom examined a work which has given us more satisfaction, both in its design and execution, or which we can more strongly recommend as calculated to afford sound knowledge of its subject in a clear and attractive form.

The bulky Vegetable Chemistry of Dr. Thomson professes to lay before the British chemical public a pretty full view of the present state of this department of the science. To us, however, the mode in which this task has been accomplished is far from being satisfactory, and we hardly think the work worthy of the distinguished author's reputation. It contains an immense mass of materials, but they are ill digested. All sorts of analyses, good, bad, and indifferent, are brought together, without any proper directions to the student as to their respective value; and he who is not previously acquainted with the name and character of the chemist whose results are set forth, will be quite in the dark as to the probability of their accuracy. To the advanced student the work will have much value, as comprising a vast amount of materials which he would otherwise have had to seek in many scattered sources: it is not suited to the beginner.

---

